# Reviews and Book Notices

**Published:** 1881-05

**Authors:** 


### Reviews and Book Notices.

---

ARTICLE XIII.—A MANUAL OF THE PRACTICE OF MEDICINE. DESIGNED FOR THE USE OF STUDENTS AND THE GENERAL PRACTITIONER. By Henry C. Moir, M.D. 12mo, pp. 454, cloth. New York: Steam Press of the Industrial School, H. O. A., 1881.

There is a want felt by the students of medical colleges, we are sorry to say, for concise manuals, or mere groupings and tabulatings of the most important facts known in medicine. By their help, any school boy, and some who have never been to school, (provided they can read) can commit to memory enough definitions and opinions to become a medical gentleman in a couple of four-months terms. The book under notice supplies such a want better than any other one that we know. Taking for instance, "Croupous (Lobar) Pneumonia. Stages: *First*, engorgement; *second*, red hepatization; *third*, gray hepatization. Etiology. *Predisposing causes:* Age (most common between twenty and forty); sudden change in temperature; poverty; intemperance. *Exciting causes:* Exposure to cold or wet (as chilling of the feet or body); special diseases (as exanthematous fevers, uræmia, pyæmia, septicæmia, alcoholism, etc.) Traumatism." Follow thirteen rational symptoms in order, and fifteen physical ones. "Differential diagnosis: From cerebral disease (which ?) and cerebro-spinal-meningitis (in children); from pleurisy in all its forms; phthisis (second stage; from capillary bronchitis, and pulmonary œdema." In the treatment of typhoid fever: "*Quinine in large doses,* 30 *to* 40 *grains within two hours, or* 10 *grains every half hour for four doses,* etc. This is also advised in small-pox, whenever the temperature rises above

104°. As a rule, however, we must say that this book embraces a "résumé of such standard works as those of Niemeyer, Roberts, Loomis, Da Costa, Bristowe, Hartshorne, and others," and is not quite devoid of value, especially to students waiting in the antechamber of the *green room.* It is beautifully printed, with *leading* and entirely blank pages, every here and there. In enumerating symptoms, each one has a separate line, and 45 pages, devoted to prescriptions, contain 26 pages of printed matter only. So that the whole work might have advantageously appeared in a pocket-book of 200 pages, with quite a saving of money to the needy student.

Article XIV.—A Treatise on the Principles and Practice of Medicine; Designed for the Use of Practitioners and Students of Medicine. By Austin Flint, m.d., Professor of the Principles and Practice of Medicine and of Clinical Medicine in the Bellevue Hospital Medical College; etc. Fifth Edition revised and largely re-written. 8vo. pp. 1150, half Russia. Philadelphia: Henry C. Lea's Son & Co.; 1880. Jansen, McClurg & Co., Chicago.

This favorite old friend of medical students and practitioners, now appears with such valuable improvements, that it will certainly resume its place at the head of the "manuals" most used in this country. The want for a revised edition of this book has been felt in many medical colleges, especially since 1874, when the first volume of Ziemssen's Cyclopædia was published. A more valuable work is not found in the United States, while none of the foreign books are well adapted to our present wants.

From the Preface: "The labor of the revision has been shared by Dr. William H. Welch, Lecturer on Pathological Histology in the Bellevue Hospital Medical College. * * It may be claimed for the present edition that the eliminations, substitutions and additions render it essentially a new work * * the author ventures to hope that it may be found to represent fairly the existing state of the science of medicine with respect to the subjects of which it treats, and to reflect the views of those who exemplify in their practice the present stage of the progress of medical art." In the chapter on Phthisis, a disease which has been so carefully studied and so successfully treated by the author, he says that he never met an inebriate who owed it to the medical use of alcoholics in that affection. As a valuable remedy in the treatment of whooping cough, oxalate of cerium has been forgotten. A feature of this manual is the variety of useful modes of treating diseases which are found to fill the expectations of the most sanguine, and open a wide field to clinical study and experiments. H. D. V.

ARTICLE XV.—A MANUAL FOR THE PRACTICE OF SURGERY. By Thomas Bryant, F.R.C.S., Surgeon to, and Lecturer on Surgery at, Guy's Hospital; etc. Third American, from the Third Revised and Enlarged English Edition. Edited and Enlarged for the use of the American Student and Practitioner, by John B. Roberts, A.M., M.D., Lecturer on Anatomy and on Operative Surgery in the Philadelphia School of Anatomy; etc. With 735 Illustrations. Large 8vo, half Russia, pp. 1005. Philadelphia: Henry C. Lea's Son & Co.; 1881. Jansen, McClurg & Co., Chicago.

For medical students this is one of the most valuable works on surgery, embodying as it does the views and experience of so eminent a surgeon as Mr. Bryant. It will even answer the purpose of many practitioners, but not as well as the classical work of S. D. Gross, or the late treatise of Agnew. American surgery has, since the rebellion, acquired a national character which should not be overlooked. Fractures and dislocations are not treated at sufficient length, less than 100 pages being consecrated to that important part of practical surgery, in such a manner that a supplementary volume, like that of Hamilton, would be needed to complete the work. The illustrations are all that could be desired. The additions of the American editor are most practical, and one wishes that they were more numerous. The half Russia leather binding of this and other volumes just published by the same firm, is very pretty, and will advantageously compare with all other bindings on the shelves of every library; though they have a disadvantage which they partly share with cloth covers, and are permanently injured if their sides are exposed to rain or water. H. D. V.

ARTICLE XVI.—STUDENT'S POCKET MEDICAL LEXICON: Giving the Correct Pronunciation and Definition of all Words and Terms in General Use in Medicine and the Collateral Sciences, the pronunciation being plainly represented in the American phonetic alphabet. With an Appendix containing a list of Poisons and their Antidotes; Abbreviations used in Prescriptions; and a Metric Scale of Doses. By Elias Longley, author of a "Pronouncing Vocabulary of Geographical and Personal Names;" "Eclectic Manual of Phonography;" and a Series of Phonetic School Books. 16mo, cloth, pp. 304. Philadelphia: Lindsay & Blakiston; 1879.

This dictionary contains about the same number of words as "Wood's Medical Lexicon," which is better known to students. The introduction of twenty new letters or signs, borrowed from various alphabets, seems more an incumbrance than an improvement in the pronouncing part. Besides the excellence of its typography, it has no special merit of which we are aware, and does not fill the want of a lexicon, wherein definitions of many new words in medicine should be found. H. D. V.

ARTICLE XVII.—AN INDEX OF COMPARATIVE THERAPEUTICS, WITH TABLES OF DIFFERENTIAL DIAGNOSIS, DOSE LIST, ETC. By Samuel O. L. Potter, M.D., President Milwaukee Academy of Medicine, etc. Chicago: Duncan Brothers; 1880.

"The object aimed at in this book is to present the therapeutics of the two great medical schools in the manner best adapted to comparative study and quick reference." The remedies recommended by some of the most eminent teachers in both schools (allopathic and homœopathic) are arranged in parallel columns for convenient references. The work is not exhaustive; only the outlines of the subject are given. It contains no new information. The chief interest of the book is the comparison between regular and homœopathic therapeutics. B. W. G.

